# A generalized simulation development approach for predicting refugee destinations

**DOI:** 10.1038/s41598-017-13828-9

**Published:** 2017-10-17

**Authors:** Diana Suleimenova, David Bell, Derek Groen

**Affiliations:** 1 Brunel University London, Department of Computer Science, London, UB8 3PH United Kingdom; 2 University College London, Centre for Computational Science, London, WC1H 0AJ United Kingdom

## Abstract

In recent years, global forced displacement has reached record levels, with 22.5 million refugees worldwide. Forecasting refugee movements is important, as accurate predictions can help save refugee lives by allowing governments and NGOs to conduct a better informed allocation of humanitarian resources. Here, we propose a generalized simulation development approach to predict the destinations of refugee movements in conflict regions. In this approach, we synthesize data from UNHCR, ACLED and Bing Maps to construct agent-based simulations of refugee movements. We apply our approach to develop, run and validate refugee movement simulations set in three major African conflicts, estimating the distribution of incoming refugees across destination camps, given the expected total number of refugees in the conflict. Our simulations consistently predict more than 75% of the refugee destinations correctly after the first 12 days, and consistently outperform alternative naive forecasting techniques. Using our approach, we are also able to reproduce key trends in refugee arrival rates found in the UNHCR data.

## Introduction

Global forced displacement has reached record levels. In 2017, 65.6 million people were forcibly displaced worldwide, a number which includes 22.5 million refugees^1^. Common causes of forced migration include push and pull characteristics, such as the present social, political, and economic conditions of migrants’ origin and potential destination, as well as intervening characteristics between these two locations^2,3^. Migration is a complex phenomenon and the push-pull characteristics can be insufficient to explain forced migration^4^. Several groups identified sets of other causal factors that lead to forced displacement, including conflicts, ethnic or religious differences, and existential obstacles such as severe ecological decline^5,6^.

Previous studies have shown that the influence of these causal factors can be determined using migration flow models. For instance, Shellman and Stewart^7^ investigated Haitian migration to the United States using an early warning model of forced migration and predicted risk factors, such as civil violence, economic conditions and external interventions, that forced people to migrate. Similarly, Martineau^8^ used an early warning model to predict which countries have the potential to create refugees. However, existing early warning models of forced migration focus on understanding the causes^9^ and are not as successful in predicting refugee movements as in predicting natural disasters^10–12^. Moreover, they lack the accuracy and flexibility to accommodate the context changes that lead to large-scale refugee movements^13^. As a result, there are relatively few appropriate models for predicting refugee movements^14,15^.

Computational models have been widely applied to study migration processes^16^. Moreover, they have the potential to contribute to a better understanding of refugee movement patterns, and to inform, predict and fulfil gaps within forced migration estimations^17^. In particular, computational models could be applied interactively to assist governments and organisations in estimating where and when refugees are likely to arrive^18^, and which camps are most likely to become full in the short term. Simulating refugee movements also has potential due to its reduced ethical burden, which normally impedes empirical analysis, and the possibility to derive causal relations^17^.

Agent-based modelling (ABM) is a popular simulation approach that can explicitly model social interactions and networks emerging from it. Hence, ABM is becoming a prominent method for population and migration studies (e.g.,^17,19–21^). In addition, it is popular due to its decentralized approach^22^, which allows a heterogeneous mix of many agents to act and interact autonomously, leading to emergent behaviours in the system at higher levels^22–24^. ABM is especially suitable for modelling active objects, such as individuals, animals or products, in relation to time, events or behaviour^25^, and it has been applied to model problems ranging from small-scale behavioural dynamics to large scale migration simulations^26,27^.

However, a few important challenges have been identified within the ABM community. For instance, there is an ongoing debate on whether prediction should be a major purpose for ABMs^28^, or whether explaining and illuminating problems should be a priority^29^. Specifically for migration studies, Klabunde and Willekens^22^ identify major challenges in both the definition of decision-making theories and the selection of empirical evidence for model validation.

ABMs are already used in a wide range of refugee-related settings, such as disaster-driven migration which incorporate changes in climate and demographics^30^. For example, Hassani-Mahmooei and Parris^31^ analysed the influence of climate change on migration in Bangladesh while Kniveton *et al*.^19,32^ developed an ABM to simulate climate migration in Burkina Faso between 1970–2000 and to predict future migration flows to 2060. Additionally, Anderson *et al*.^33,34^ suggested an ABM for refugee communities to inform policy decisions for governments and other organisations. The German armed forces developed an ABM to understand interactions and behaviour of refugees with military groups in a refugee camp environments^20^. In the context of predicting and forecasting refugee movements, Sokolowski and Banks^35^ developed an ABM Environment Matrix that can be used to accurately represent irregular migration movements using simulation. Similarly, Latek *et al*.^36^ build a multi-agent model that predicts the Syrian conflict characteristics and investigated potential conditions and outcomes of the conflict. Hattle *et al*.^37^ examined the Syrian refugee flows to European countries using ABM and discussed possible policy recommendations on distributing humanitarian resources amongst potential refugee hosting countries. Several groups also applied ABM to capture local aspects, such as networks, group formation and travel distance in the refugee crisis and stress the importance of computational modelling for migration predictions^21,38,39^.

In this work, we present a generalized simulation development approach (SDA) to predict the distribution of refugee arrivals across camps, given a particular conflict situation. Our SDA has six phases, and is partially based on the notion of the Simplified Simulation Development Process, presented by Heath *et al*.^40^. It encompasses the formulation of the problem (phase 1), the translation into a computer model (phase 2,3 and 4) and the operational validation (phase 5 and 6). The conceptual validation does not pertain a specific phase, as we present a conceptual model that can be readily adopted as part of the SDA.

**Figure 1:**
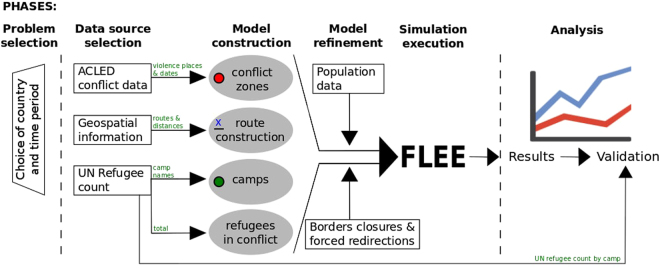
Simulation development approach for predicting the distribution of refugee arrivals across camps.

Our main reason to develop a full SDA, in contrast with merely proposing a simulation model design, is the need for organizations to facilitate rapid simulation *development* when a conflict occurs. In conflict situations, a model design alone would contain too little information to facilitate rapid development, as such development activities inevitably involve the selection of data sources, the extraction and conversion of data, as well as the validation of simulation predictions against empirical data. We provide a diagrammatic overview of our SDA, and its six phases, in Fig. 1.

Here, in the first phase, we select a country and time period of a specific conflict which resulted in large scale forced migration. In the second phase, we obtain relevant data to the conflict from three data sources: the Armed Conflict Location and Events Database (ACLED, http://www.acleddata.com/data/acled-version-7-1997-2016/acleddata.com)^41^, the UNHCR database (http://data2.unhcr.org/en/situationsdata2.unhcr.org), and the Bing Maps platform (https://www.bing.com/mapsbing.com/maps). We use ACLED to obtain the locations and dates of battles that have taken place in the conflict, and the UNHCR database to obtain the number of refugees in the conflict, as well as the camp locations and capacities. We rely on the Bing Maps platform to obtain locations of major settlements and routing information between the various camps, conflict zones and other settlements. We provide a detailed description of the data collection procedure in the Methods section.

**Figure 2:**
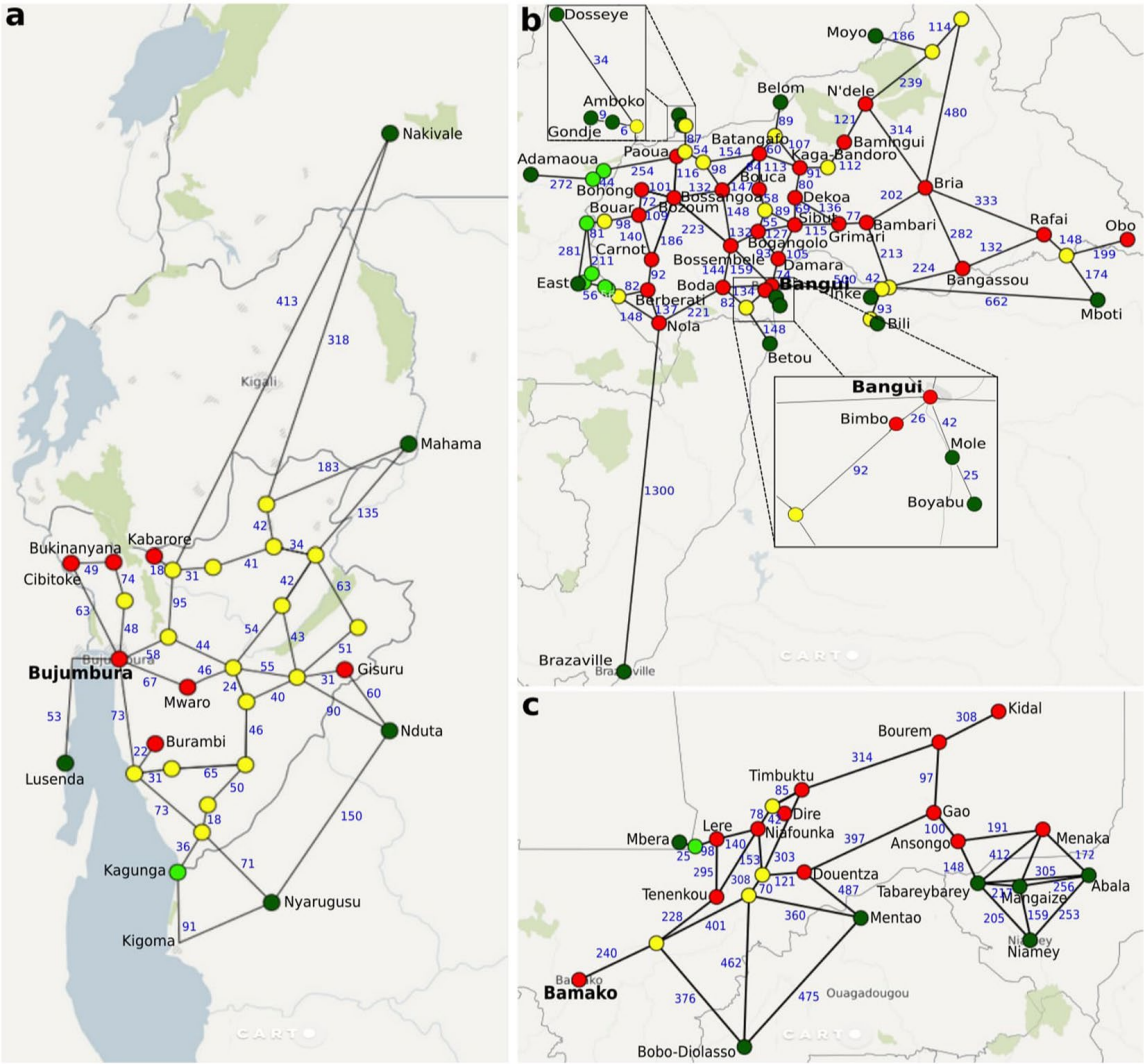
Overview of geographic network models for (**a**) Burundi, (**b**) Central African Republic and (**c**) Mali. Models contain conflict zones (red circles), refugee camps (dark green circles), forwarding hubs (light green circles) and other major settlements (yellow circles). Interconnecting roads are given in a simplified straight-line representation, with adjacent blue numbers used to indicate their length in kilometres. Background maps are courtesy of https://carto.comcarto.com created using OpenStreetMap data that is further modified with the use of https://inkscape.org/en/release/0.91Inkscape0.91.

In the third phase, we construct our initial simulation model using these data sets, and create among other things a network-based ABM model. We present the three network-based ABM models, one for each conflict we seek to model, in Fig. 2, while we present our detailed assumptions in the Methods section and provide our source data as part of Supplementary Note 2. Once we have constructed the initial mode, we refine it as part of the fourth phase. Here, we manually extract population data to help determine where refugees flee from (see Methods section for details), as well as information on border closures and forced redirections of refugees (see Supplementary Note 2).

The fifth phase involves the main simulation, which we run to predict, given a total number of refugees in the conflict, the distribution of refugees across the individual camps. We run our simulations using the FLEE simulation code. FLEE is optimised for simplicity and flexibility and provides a range of scripts to handle and convert refugee data from the UNHCR database. As part of this work, we publicly release the FLEE code, as well as all our input and output data sets, under a BSD 3-clause license (see the subsections on Code Availability and Data Availability in the Methods section). Once the simulations have completed, we analyse and validate the results against the full UNHCR refugee numbers as part of the sixth phase (see Results section for several examples).

To showcase the added value, and generalized nature, of our SDA, we apply it to model three refugee crises in African countries. These crises include the 2015–2016 civil war in Burundi and the 2013–2016 conflict in the Central African Republic (CAR), both which to our knowledge have never been modelled before. We also model the Northern Mali conflict in 2012–2013^42,43^, which we have previously modelled in rudimentary form (see Supplementary Note 5).

These three African countries demonstrate different conflict initiation scenarios, but they all have common drivers forcing people to flee, such as political instabilities, violence and civil war. According to Turchin^44^, when countries experience long-term pressures they result in civil wars, social and political instabilities. By understanding historical data of these socio-political instabilities and political violence events, it is possible to find patterns that explain their cause and time of occurrence^45–47^. Though a full historical analysis of the three conflicts of interest is well beyond the scope of this work, we do provide a brief summary of each conflict in Supplementary Note 1. The Burundian crisis was triggered by the third-term election of President Pierre Nkurunziza in April 2015. His election triggered protests, coups and eventually a refugee crisis^48,49^. We choose to simulate this conflict from the start of the refugee crisis, around the 1st of May 2015, until the 31st of May 2016, for a period of 396 days. In CAR, the Seleka group (Muslim population) overthrew the central government, in March 2013^50^. Not long after, anti-Balaka (Christian militia groups) took over the power. Muslim and Christian communities started a long string of conflicts and violent attacks^51^. The crisis continued for several years and to capture it simulation period is 820 days from 1 December 2013 to 29 February 2016. In the case of Mali, the crisis was due to insurgent groups, who began a campaign to fight for the independence of the Azawad region. The conflict started on the 16th January 2012, when Touareg rebels began conquering settlements in Northern Mali^17^. In this case, we selected a simulation period of 300 days, from the 29th of February 2012, when the first camp registrations were recorded, until 25th December 2012, when the vast majority of refugees had been registered in the camps.

## Results

We present results from our SDA, which we applied to predict the distribution of refugees across camps in three African conflicts. For each conflict, we compare our prediction results with the UNHCR refugee camp registration data. We provide a list of the refugee camps in each conflict in Table 1.

**Table 1:** List of existing camps used in simulations.

Country	Neighbouring country	Camps
Burundi	Tanzania	Nyarugusu and Nduta
	Rwanda	Mahama
	Uganda	Nakivale
	Democratic Republic of the Congo (DRC)	Lusenda
CAR	Cameroon	East and Adamaoua
	Chad	Belom, Dosseye, Amboko,
		Gondje and Moyo
	DRC	Inke, Mole, Bili,
		Mboti and Boyabu
	Republic of the Congo (RC)	Betou and Brazaville
Mali	Mauritania	Mbera
	Burkina Faso	Mentao and Bobo-Dioulasso
	Niger	Abala, Mangaize, Niamey and Tabareybarey

**Figure 3:**
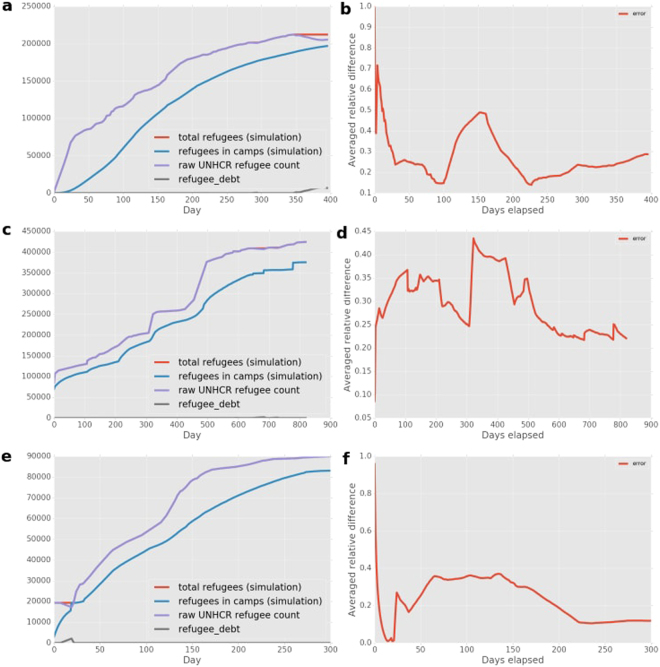
Comparison of number of refugees in camps between the simulation and the data (left column), and overview of the averaged relative difference between simulation and data (right column). The averaged relative difference across camps between simulation and data is given by the red line. We provide these comparisons respectively for (**a,b**) the Burundi simulations (top row), (**c,d**) the CAR simulations (middle row) and (**e,f**) the Mali simulations (bottom row).

We also present several error measures in Fig. 3, including an overview of the number of refugees in camps according to the simulation and the UNHCR data in (Fig. 3a,c and e) and the averaged relative difference between the simulation results and the UNHCR data (explained in the Methods Section) in Fig. 3b,d and f. The averaged relative difference is less than 0.5 after the first few days, indicating that our simulations accurately predict more than 75% of the refugee movements in absolute terms. In all our runs, the averaged relative difference is lower at later stages of the simulations, with relative differences of 0.1–0.3 or towards the end of all runs.

### Burundi

**Figure 4:**
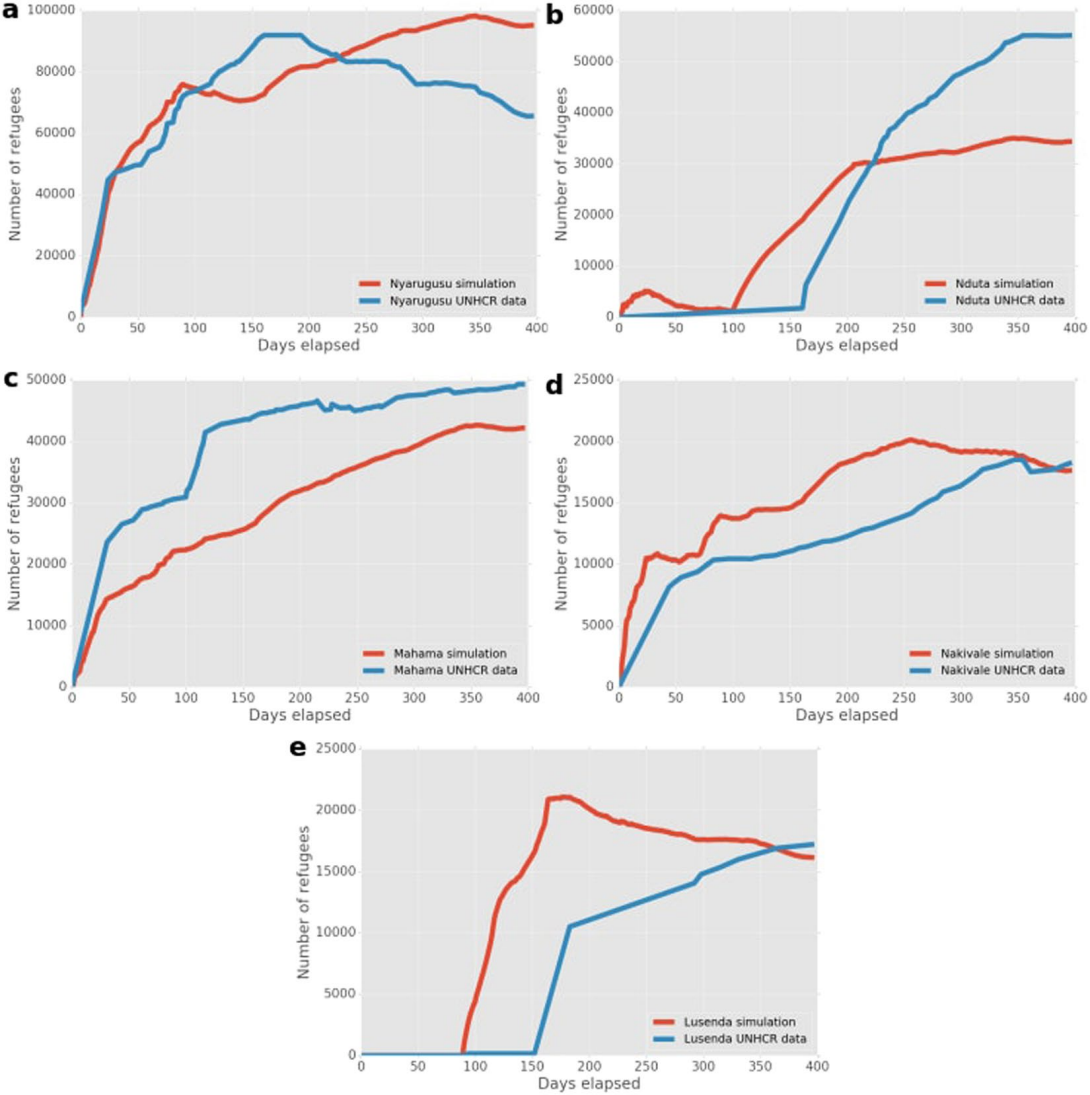
Number of refugees as predicted by our simulation and obtained from the UNHCR data for the Burundi conflict. (**a**–**e**) Graphs are ordered by camp population size, with the most populous camp on the top to the smallest one on the bottom.

We present our simulation predictions and the UNHCR refugee counts for the Burundi conflict in Fig. 4. Within the camps in Nyarugusu, Mahama and Nakivale, our simulation results accurately capture the key growth trends in refugees. Our approach does underpredict the refugee population growth in Mahama, as there is a delay in refugee arrival due to the many non-conflict settlements between Mahama and the conflict zones.

Both the Nduta and Lusenda camps opened only after the start of the period of simulation. Nduta was only established as a refugee camp on the 10th of August 2015 (day 101), after Nyarugusu became overpopulated. In the case of Nduta, our simulation shows a small population of travelling refugees at the start (when the location was not yet a camp), and a steep population increase to 30,000 during the 90 days after the camp is opened. Lusenda, which opened on day 90, quickly fills to capacity in the simulation, whereas a more gradual increase can be observed in the data. Here, the mismatch could be due to delays in the UNHCR registration process, as virtually no refugees were properly registered in the whole of DRC prior to the 30th of October 2015 (day 182).

For Burundi (Fig. 3a), our simulations contain substantially fewer refugees in camps than the UNHCR measurements for the same day. This difference is larger than in other cases and affects the averaged relative difference (Fig. 3b), primarily because Burundi is a densely populated country with a large number of settlements in the network graph. However, the difference decreases after Day 5 once substantial numbers of refugees arrive in camps in the simulation, and only increases to a peak around 0.48 on Day 151, due to a coincidence of peak mismatches in both Nyarugusu and Nduta.

### Central African Republic

**Figure 5:**
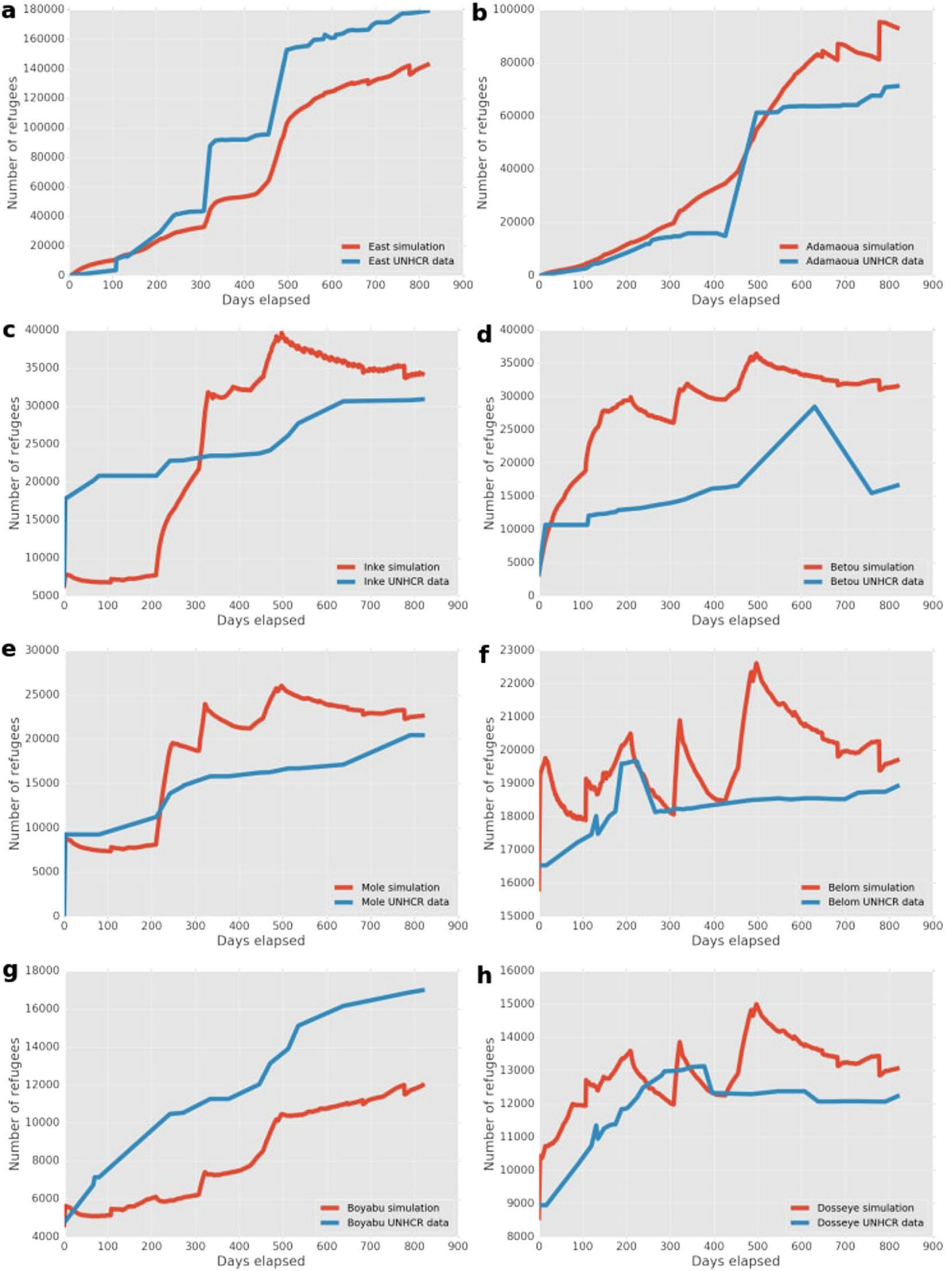
Number of refugees as predicted by our simulation and obtained from the UNHCR data for the CAR conflict. (**a**–**h**) Graphs are ordered by camp population size, with the most populous camp on the top to the smallest one on the bottom (see remaining six camps in Fig. S1).

In Fig. 5 we present the number of refugees in camps for the CAR conflict simulation. Our simulation predictions closely follow the trends observed in the data for the two largest camps, East Congo and Adamaoua. Here our simulation underpredicts the total refugee population in East Congo by about 35,000 (~20%), and overpredicts the population in Adamaoua by about 23,000 (~30%).

The camps in DRC (Inke, Mole, Boyabu and Mboti) were subject to border closures between CAR and DRC from the 5th of December 2013 (simulation day 4), until the 30th of June 2014 (day 211, see Supplementary Note 2 for details). This is reflected by a period of relatively stable refugee populations both in the simulation and in the data. Bili also is located within DRC, but was established only after the border was reopened.

The predicted refugee counts in the Chad camps (Amboko, Belom, Dosseye and Gondje) are in close agreement with the data, except that large fluctuations occur during the simulation after the border closure on the 12th of May 2014 (day 163). At this time all the camps are close to full occupancy, which results in refugees moving from between the camps and the city of Gore, a city in Chad which lies in close proximity to the camps.

The Betou camp in Congo is an another example of a camp close to the conflict areas, and it also fills up quickly in the simulation. The Brazaville location is far removed from the conflict zone, and here our simulation underpredicts the refugee population. It could be that the size of the city of Brazaville may increase its attractiveness as a refugee destination. We did not incorporate this factor in the runs presented here, but we do wish to examine it in future simulation studies.

In the CAR situation (Fig. 3c and d) the mismatch in the number of refugees remains relatively small, while the averaged relative difference fluctuates around 0.3. The jump in error around Day 300 is largely due to a sudden large increase in refugees in East Cameroon at that time, according to the UNHCR data.

### Mali

**Figure 6:**
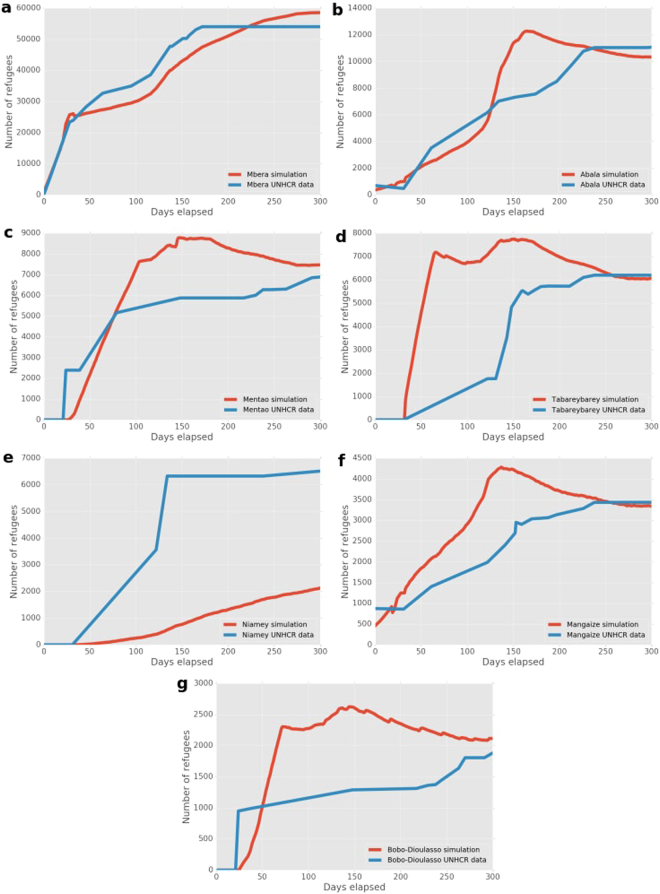
Number of refugees as predicted by our simulation and obtained from the UNHCR data for the Mali conflict. (**a**–**g**) Graphs are ordered by camp population size, with the most populous camp on the top to the smallest one on the bottom.

In Fig. 6 we present the number of refugees in camps around Mali over the 300 day simulation period. Our simulation results are in close agreement with the data for the two largest camps. The maximum differences here are an underprediction of 7,000 (~18%) for Mbera around day 135, and an overprediction of about 4,500 (~60%) for Abala around day 160. Tabareybarey, Niamey, Mentao and Bobo-Dioulasso were established once the conflict was already underway. Tabareybarey and Niamey camps have refugees for simulation and data from day 30, whereas the camps in Burkina Faso, Mentao and Bobo-Dioulasso, reopened their previously closed borders on the 1st of April 2012 (day 32).

The simulation predicts a fast-paced growth of refugee population for both Mentao and Bobo-Dioulasso, while the data features a sudden spike in refugee arrivals around day 30 in these camps. The simulation predictions for Mangaize in Niger are in line with the data, though slightly higher. The large inflow early in the simulation is primarily due to the close proximity of Mangaize to one of the early conflict zones (Menaka). Our simulation results do not accurately match the data for Tabareybarey and Niamey. Niamey is not directly connected to regions in Mali, due to two other refugee camps being located along the way. However, Niamey is a large capital city (like Brazaville in the CAR simulation) which may be the reason why more refugees choose that destination than our simulation predicts. In general, our predictions overestimate the refugee inflow into the three border camps in Niger. An important cause here may be the presence of partial restrictions for crossing the Niger border during the conflict^17^.

In the Mali situation (Fig. 3e and f) we see a large but decreasing mismatch at the very start of the simulation. This is because the Fassala camp is technically not defined as a camp within our simulation, as refugees were already redirected from Fassala to Mbera from the start of the simulation period. However, Fassala is considered to be a camp according to the data. After Day 30, the number of refugees in camps in the simulation is relatively close to the reported number, and the averaged relative difference remains relatively constant.

### Comparison with naive prediction models

To our knowledge, there are no other prediction techniques that have been previously applied in this setting. However, it is possible to perform naive predictions, extrapolating future behaviour from historical data, after a conflict has started.

To measure the added value of our prediction approach, we here present a comparison of our method against a set of naive prediction models. We compare the accuracy of our method by obtaining the Mean Absolute Scaled Error (MASE) relative to the six other techniques (see methods section for definition). The MASE was first proposed by Hyndman *et al*.^52^, and is well suited to quantify simulation accuracy due to its scale invariant nature and the fact that it symmetrically penalizes overestimations and underestimations. In addition, the MASE is straightforward to interpret: in our case its value is less than one if our prediction approach has a smaller error, while its value is more than one if the selected naive technique as a smaller error.

For comparison purposes, we have created three different types of naive models, all of which rely on some section of historical data to extrapolate values in the future. While our simulation approach can be used from Day 1 to provide a prediction of camp refugee populations, we can only apply naive models after a number of days have elapsed. This is because naive models extrapolate from past data; and such data can only be acquired after the conflict has started and refugees have departed.

In this section we compare our approach, as described in the main paper, against naive model predictions that take place respectively 7 days, and 30 days after the starting date of the respective simulation periods. We argue that a week is required to obtain sufficient data to apply any kind of meaningful extrapolation. However, naive models that require more than a month before they can be applied are arguably of little use, as many of the initial refugee movements have already taken place by then (particularly in the case of the Burundi conflict). It should be noted that the collection of refugee registrations is by no means an instantaneous process, and any time overhead in obtaining such would further delay the application of these naive models.

For each refugee camp location in each conflict, we have applied the following three types of naive prediction:

- ${0}^{th}$ **order (flat) extrapolation**: Here we take the refugee count on either day 7 or day 30 in each camp, and assume that this number does not change over time.
- ${1}^{st}$ **order (sloped) extrapolation**: Here we take the refugee count on either day 7 or day 30, as well as the registration count on day 0. We then linearly extrapolate future values in time from these two registration counts.
- **Extrapolation by ratio (fraction)**: Here we take the refugee fraction in a given camp, which we calculate by dividing the refugee count in a given camp, on either day 7 or day 30, by the total number of refugees across all camps on that same day. We then forecast refugee counts in each camp by assuming that this refugee fraction remains constant over time, and predict future value by taking that fixed fraction of the total refugee population (which is a known quantity in our setting) over time.

We present the results from our comparison in Table 2. In all cases, our prediction approach results in a lower averaged relative difference than the naive prediction models. We obtained MASE scores of 0.0639-0.942 (Burundi), 0.0367-0.705 (Central African Republic), and 0.116-0.513 (Mali).

**Table 2:** Comparison of our prediction approach against six naive models for each of our three conflict simulations.

**Run name**	MASE 7 Day			MASE 30 Day		
	flat	slope	fraction	flat	slope	fraction
Burundi	0.279	0.144	0.942	0.443	0.0639	0.791
CAR	0.585	0.705	0.452	0.639	0.0367	0.341
Mali	0.23	0.127	0.503	0.314	0.116	0.513
Weighted average	0.453	0.473	0.598	0.542	0.0544	0.491

Simulations were run with the same settings as those presented in the main paper. We report on the Mean Averaged Scaled Error for each of the naive models in columns 2 to 7. Here values below 1 indicate that forecasts using our prediction approach resulted in a smaller averaged relative difference than those relying on that specific naive model. In the bottom row we provide a weighted average of the MASE score across the three conflict simulations, with the weightings based on the maximum number of refugees in each conflict (205445 for Burundi, 424496 for CAR, and 89991 for Mali). Please refer to the Methods Section for details on the six naive models.

## Discussion

We have presented a generalized simulation development approach (SDA) for predicting the distribution of incoming refugees across destination camps. Accurate predictions can help save refugee lives, as it helps governments and NGOs to correctly allocate humanitarian resources to refugee camps before the (often malnourished or injured) refugees themselves have arrived. To our knowledge, we are the first to attempt such predictions across multiple major conflicts using a single simulation approach.

Using our approach, we have reproduced the key refugee movement patterns in each of the three conflicts and correctly predicted at least 75% of the refugee movement destinations in all these conflicts after the first 12 days. In the Burundi conflict, our approach correctly predicts the largest inflows in Nyarugusu, Mahama and Nakivale during the early stages of the conflict. In CAR, our prediction approach correctly reproduces the growth pattern in East Congo, as well as the stagnation of refugee influx in the Chad camps. In the case of Mali, our predictions accurately capture the trends in the data for both Mbera and Abala, which together already account for ~75% of the refugee population. Our results are insensitive to most simulation parameter changes, with the notable exception that increasing the probability for refugee agents in non-conflict/non-camp locations actually results in a further reduced error (see Methods section for a summary and Supplementary Note 4 for a detailed discussion regarding this).

As a result of conducting this study, we discovered several important issues and limitations. For example, our model omits a range of factors which are considered important according to the empirical literature, but for which we could not find accurate and tractable means to convert empirical conclusions to simulation parameters. In some cases such as GDP and presence of existing conflicts, the significance of these factors has been confirmed on a country-by-country level but not on a city-by-city level^3,53^. In other cases, such as religion and ethnicity, we simply did not find reliable statistical information on a local level for these conflicts. Some parameters, such as the level of knowledge of refugee agents about the surrounding region, were found to have little effect on the simulation results beyond being aware of adjacent locations (see Table S5). The obtained averaged relative difference also changes little when we adjust maximum movement speed of refugees to values less or more than 200 kilometres per day (see Table S4).

In general, empirical data collection during these conflicts is very challenging, in part due to the nature of the environment and in part due to the severe and structural funding shortages of UNHCR emergency response missions. Both CAR and Burundi are among the most underfunded UNHCR refugee response operations, with funding shortages of respectively 76 and 62%^54^. More funding for these operations are bound to save human lives, have the side benefit of providing more comprehensive empirical data, and thereby enable the validation of more detailed prediction models.

An additional important element that is absent in all our data sources is indications of the level of data-related uncertainty. Knowledge of this level of uncertainty would allow us to accurately quantify how uncertainty from the source data affects the overall outcome of our simulations, the quality of our validation tests, and the performance of our approach versus that of naive models.

Yet, important steps have been made in recent years, as the combination of a conflicts database^41^, a public UNHCR refugee data repository and a sophisticated mapping platform enabled us to do this work. And given the increasing effort in collecting refugee data, and increasing recognition for data science, we are confident that future research efforts on modelling refugee movements will be accelerated by ongoing advances in data collection.

## Methods

### Processing input and validation refugee data

To obtain our input data, we took the following steps. First, we selected three conflicts that featured on data2.unhcr.org (accessed June 2016) and manually obtained the refugee registration data for each camp from the website in comma separated value (CSV) format. We refined the data by interpolating linearly between data points and calculated the total refugee count by aggregating the (interpolated) registrations for each of the camps. The source data includes level 1 refugee registrations and, after certain dates, level 2 registrations. As level 1 registrations are known to result in overestimations of refugee count, we scaled down these values such that the last data point using level 1 refugee registrations matches the first data point using level 2 registrations. We exclude Internally Displaced People from the model, as there is a lack of systematic data providing their exact destinations in our scenarios.

We obtain conflict locations from the ACLED database, omitting settlements with less than 10,000 inhabitants, and noted the start date of any event labelled as “battle” during the simulation period. Locations are labelled as a conflict zone as soon as such an event has occurred. All conflict locations are assigned a population based on the latest census data.

### Constructing the network graphs

We provide detailed network graphs in Fig. 2. We selected locations by combining our ACLED conflict locations and UNHCR camp locations with major settlements that reside en-route between these locations. Locations are interconnected with links in cases where we noticed the presence of roads in Bing Maps, the length of the link (in km) was then estimated using the Bing route planner for cars. In cases where obvious shorter routes were visible, we dragged the Bing marker to force the software to calculate this shorter route. To retain the simplicity of our model, and to reflect the frequent occurrence of direct redirections of refugees to camps, we directly connected refugee camps to the nearest location in the country of conflict. In some cases, we added “forwarding” locations, where refugees are automatically rerouted to a camp, or opened camps after the start of the simulation, following descriptions in UNHCR reports (see Supplementary Note 2 for details). We also removed links when border closures were reported by the UNHCR, and added a link after the start of the simulation when a border opening was reported (see Supplementary Note 2 for details).

### Choosing simulation parameters and assumptions

We provide a flowchart of the key elements in our simulation algorithm in Supplementary Note 6. Each step of the simulation represents one day. During each step, we insert a number of refugees into the simulation based on the daily increase in the total refugee registration count from the UNHCR data. These refugees are inserted in their location of origin, which is one of the conflict locations (as obtained from the ACLED database, see section “Processing input and validation refugee data”). The exact location is picked among all conflict zones, where the likelihood of each conflict zone being selected is proportional to its population. The population of a location is decremented by one each time a refugee agent is created. Because we insert refugees in conflict zones on the day of camp registration and refugee travel is non-instantaneous, our simulation approach normally results in an under-prediction of the number of refugees. To correct for this, we multiply the refugee populations in each of the camps by *N*_*data*_,_*all*_/*N*_*sim*_,_*all*_, where *N*_*data*_,_*all*_ is the total refugee count for the conflict on a given day according to the UNHCR data. In our setting, this is a known quantity, as we are predicting the distribution of refugees across camps, given this total refugee count. *N*_*sim*_, _*all*_ is the total number of refugees in camps according to the simulation on that same day. We discuss and measure the effect of using this correction in Supplementary Note 3. We did not rescale our output when comparing the number of agents in the simulation and the data (see Fig. 3a,c and e). Decreases in UNHCR refugee registrations increment a “refugee debt” variable, which first needs to be compensated by subsequent registration increases before additional agents are again inserted in the simulation (i.e., we do not delete agents).

During each step, a refugee agent can traverse zero, one or more links. The probability of traversing a link is determined by the move chance, which we initially set at 1.0 for refugees in transit between locations, 1.0 for refugees in conflict locations, 0.001 for those in refugee camps, and 0.3 for those in all other locations. As we could not find empirical evidence supporting these parameters, we initially chose these parameters based on our intuitions, and performed our main simulations using these initial choices (i.e., we did not optimize our parameter choices to minimize the error, as we believe such parameter fitting could reduce the applicability of our approach to other conflict situations). After the main run was performed, we analyzed the sensitivity of each of these parameters (see Supplementary Note 4 for details). To summarize this analysis, we found that our results are insensitive to the conflict location move chance parameter across the full tested range, and insensitive to the refugee camp move chance for values ≤0.01 (which implies the assumption that refugees remain in a refugee camp, on average, for 100 days or more). However, our simulations results did show sensitivity to the move chance for all other locations, with higher move chances resulting in smaller validation errors, and lower move chances resulting in larger validation errors. We reflect on the implications of this parameter sensitivity in detail in Supplementary Note 4.

When an agent traverses a link (with the probability determined by the aforementioned move chance) it needs to choose one of the available paths. Path selection is done using a weighted probability function, the weight of each link being equal to the attractiveness value of the destination divided by the length of the link in kilometres. The attractiveness value of the destinations equals 0.25 for conflict zones, 1.0 for other locations in the country of conflict, and 2.0 for locations abroad. Again, these values were initially chosen based on our own intuition, with the sensitivity being analyzed after the main run was performed (see Supplementary Note 4). In the case of these two parameters (attractiveness value for camps, and for conflict zones), we found that none of these parameters had a signification effect on the accuracy of our simulation. We also assumed that refugees travel no more than 200 km/day, and likewise found our simulation has low sensitivity to higher travel limits (see Supplementary Note 4), though our error increases if we choose much lower travel limits. If a refugee reaches the end of a link but has travelled less than 200 km on that day, then a new move chance calculation (and possible move) is performed. In traversing between locations, refugees take major roads, which are shortest journey paths identified using route planners from https://www.bing.com/mapsbing.com/maps and https://www.google.co.uk/mapsgoogle.co.uk/maps

### Processing simulation output data

We calculate an averaged relative difference using the following equation:

1 $$E(t)=\frac{\sum _{x\in S}(|{n}_{sim,x,t}-{n}_{data,x,t}|)}{{N}_{data,all}}$$

Thus, the number of refugees found in each camp $x$ of the set of all camps *S* at time *t* is given by *N*_*sim*_,_*x*_,_*t*_ based on the simulation predictions, and by *n*_*data*_,_*x*_,_*t*_ based on the UNHCR data. The total number of refugees reported in the UNHCR data is given by *N*_*data*_,_*all*_. We also present comparisons to naive models using the Mean Absolute Scaled Error (MASE). We calculate the MASE score using the aforementioned averaged relative difference at each time step, as follows:

2 $$MASE=\frac{1}{T}\frac{\sum _{t=0}^{T}E(t)}{\frac{1}{T-w}\sum _{t=w}^{T}{E}_{naive}(t)}$$

Here, *T* is the full duration of the simulation, *w* is the warmup period required for the naive model to make its predictions (in our case either 7 or 30 days, depending on the model type). The averaged relative difference using the naive model compared to the validation data is given by *E*_*naive*_(*t*).

### Code Availability

We use the FLEE^17^ for our simulations, which is an agent-based modelling code written in Python with a limited feature set that is optimised for simplicity and flexibility. It is able to support simulations with 100,000 s of agents on a single desktop, and provides users with the ability to define and use their own models through a relatively straightforward API. We provide a range of functional tests to allow users to verify the consistency of the code results. FLEE also features a range of scripts to handle and convert refugee data from data2.unhcr.org, as well as an automated plotting tool for output generated by the simulation. To use the code, one requires a Python 3 interpreter, as well as the numpy, scipy and pandas Python modules. As part of this publication, we provide the version of FLEE we used to run these simulations. This version can be found at http://www.github.com/djgroen/flee-release, and is distributed under a BSD 3-clause license.

### Data Availability

All input and output data publicly available on Figshare with DOI https://doi.org/10.17633/rd.brunel.5446813.v1, under a CC-By 4.0 license.

## Electronic supplementary material

Supplementary Information

## References

[1] UNHCR. Figures at a glance. United Nations High Commissioner for Refugees. Available at: http://www.unhcr.org/uk/figures-at-a-glance.html (2017).

[2] Lee ES (1966). A theory of migration. Demography.

[3] Moore WH, Shellman SM (2007). Whither will they go? A global study of refugees’ destinations, 1965–1995. International Studies Quarterly.

[4] EASO. The push and pull factors of asylum-related migration. A literature review. European Asylum Support Office (2016).

[5] Wood WB (1994). Forced migration: Local conflicts and international dilemmas. Annals of the Association of American geographers.

[6] Kalipeni E, Oppong J (1998). The refugee crisis in Africa and implications for health and disease: A political ecology approach. Social Science & Medicine.

[7] Shellman SM, Stewart BM (2007). Predicting risk factors associated with forced migration: An early warning model of Haitian flight. Civil Wars.

[8] Martineau JS (2010). Red flags: A model for the early warning of refugee outflows. Journal of Immigrant & Refugee Studies.

[9] Bunoiu, M.-D. & Udroiu, I. Spotting trouble in migration flows: An indicator-based early warning model. In Iancu, N. & Chiru, I. (eds) *Proceedings of the XXIst International Conference. Intelligence in the Knowledge Society*, 95–104 (Bucharest, 2016).

[10] Schmeidl S, Jenkins JC (1998). The early warning of humanitarian disasters: Problems in building an early warning system. International Migration Review.

[11] Schmeidl, S. The early warning of forced migration: State or human security? In *Refugees and Forced Displacement: International Security, Human Vulnerability, and the State*, 130–155 (United Nations University Press, Tokyo, 2003), newman, e. and selm j.v. (ed.) edn.

[12] Birkmann J, Chang Seng D, Setiadi N (2013). Enhancing early warning in the light of migration and environmental shocks. Environmental Science and Policy.

[13] Lopez-Lucia, E. Early warning models for irregular migration. Helpdesk Research Report. GSDRC Applied Knowledge Services (2015).

[14] Edwards S (2008). Computational tools in predicting and assessing forced migration. Journal of Refugee Studies.

[15] Disney, G., Wiśniowski, A., Forster, J. J., Smith, P. W. F. & Bijak, J. Evaluation of existing migration forecasting methods and models. Report for the Migration Advisory Committee: Commissioned research. ESRC Centre for Population Change, University of Southampton (2015).

[16] Willekens, F. *Migration flows: Measurement, analysis and modelin*g, 225–241, 10.1007/978-94-017-7282-2_11 (Springer Netherlands, Dordrecht, 2016).

[17] Groen D (2016). Simulating refugee movements: Where would you go?. Procedia Computer Science.

[18] Perez Estrada, L. E., Groen, D. & Ramirez-Marquez, J. E. A serious video game to support decision making on refugee aid deployment policy. *Procedia Computer Science* **108**, 205–214, 10.1016/j.procs.2017.05.112 International Conference on Computational Science, ICCS 2017, 12–14 June 2017, Zurich, Switzerland (2017).

[19] Kniveton D, Smith C, Wood S (2011). Agent-based model simulations of future changes in migration flows for Burkina Faso. Global Environmental Change.

[20] Johnson, R. T., Lampe, T. A. & Seichter, S. Calibration of an agent-based simulation model depicting a refugee camp scenario. In *Proceedings of the 2009 Winter Simulation Conference (WSC)*, 1778–1786 (2009).

[21] Sokolowski, J. A., Banks, C. M. & Hayes, R. L. Modeling population displacement in the Syrian city of Aleppo. In *Proceedings of the 2014 Winter Simulation Conference*, 252–263 (2014).

[22] Klabunde A, Willekens F (2016). Decision-making in agent-based models of migration: State of the art and challenges. European Journal of Population.

[23] Castle, C. J. & Crooks, A. T. Principles and concepts of agent-based modelling for developing geospatial simulations. Centre for Advanced Spatial Analysis, University College London (2006).

[24] Crooks A, Castle C, Batty M (2008). Key challenges in agent-based modelling for geo-spatial simulation. Computers, Environment and Urban Systems.

[25] Borshchev, A. & Filippov, A. From system dynamics and discrete event to practical agent based modeling: Reasons, techniques, tools. In *Proceedings of the 22nd International Conference of the System Dynamics Society*, vol. 22 (2004).

[26] Macal CM, North MJ (2010). Tutorial on agent-based modelling and simulation. Journal of Simulation.

[27] Macal CM (2016). Everything you need to know about agent-based modelling and simulation. Journal of Simulation.

[28] Elsenbroich C (2012). Explanation in agent-based modelling: Functions, causality or mechanisms?. Journal of Artificial Societies and Social Simulation.

[29] Epstein, J. M. Why model? *Journal of Artificial Societies and Social Simulation* **11** (2008). 10.18564/jasss.5083 PMC10210545 37235175

[30] Entwisle, B. *et al*. Climate shocks and migration: An agent-based modeling approach. *Population and Environment* 1–25 (2016). 10.1007/s11111-016-0254-y PMC5004973 27594725

[31] Hassani-Mahmooei B, Parris BW (2012). Climate change and internal migration patterns in Bangladesh: An agent-based model. Environment and Development Economics.

[32] Kniveton DR, Smith CD, Black R (2012). Emerging migration flows in a changing climate in dryland africa. Nature Climate Change.

[33] Anderson, J., Chaturvedi, A., Lengacher, D. & Cibulskis, M. Modeling the health of refugee camps: An agent-based computational approach. In *19th IEEE Symposium on Computer-Based Medical Systems (CBMS’06)*, 641–645 (2006).

[34] Anderson J, Chaturvedi A, Cibulskis M (2007). Simulation tools for developing policies for complex systems: Modeling the health and safety of refugee communities. Health care management science.

[35] Sokolowski, J. A. & Banks, C. M. A methodology for environment and agent development to model population displacement. In *Proceedings of the 2014 Symposium on Agent Directed Simulation* (2014).

[36] Łatek, M. M., Rizi, S. M. M. & Geller, A. Verification through calibration: An approach and a case study of a model of conflict in syria. In *Proceedings of the 2013 Winter Simulation Conference: Simulation: Making Decisions in a Complex World*, 1649–1660 (2013).

[37] Hattle A, Yang KS, Zeng S (2016). Modeling the Syrian refugee crisis with agents and systems. The UMAP Journal.

[38] Collins, A. J. & Frydenlund, E. Agent-based modeling and strategic group formation: A refugee case study. *Proceedings of the 2016 Winter Simulation Conference* 1289–1300 (2016).

[39] Lin, L., Carley, K. M. & Cheng, S.-F. An agent-based approach to human migration movement. In *Proceedings of the 2016 Winter Simulation Conference*, 3510–3520 (2016).

[40] Heath, B., Hill, R. & Ciarallo, F. A survey of agent-based modelling practices (January 1998 to July 2008). *Journal of Artificial Societies and Social Simulation* **12** (2009).

[41] Raleigh C, Linke A, Hegre H, Karlsen J (2010). Introducing ACLED-Armed Conflict Location and Event Data. Journal of Peace Research.

[42] Lacher, W. Organized crime and conflict in the Sahel-Sahara region. Carnegie Endowment for International Peace (2012).

[43] Lecocq B (2013). One hippopotamus and eight blind analysts: A multivocal analysis of the 2012 political crisis in the divided Republic of Mali. Review of African Political Economy.

[44] Turchin, P. Modeling social pressures toward political instability. *Cliodynamics* **4** (2013).

[45] Turchin, P. Building nations after conflict. *Nature* 986–987 (2008).

[46] Turchin P (2009). Long-term population cycles in human societies. Annals of the New York Academy of Sciences.

[47] Turchin P (2012). Dynamics of political instability in the United States, 1780–2010. Journal of Peace Research.

[48] Boyce, M. & Vigaud-Walsh, F. ‘Youare either with us or against us’: Persecution and displacement in Burundi. *Refugees International* (2015).

[49] IRRI. Burundi: A country on the edge. International Refugee Rights Initiative. Available at: http://www.refworld.org/docid/57b6f9364.html (2016).

[50] Yarnell, M. Central African Republic: The spotlight is gone, the crisis continues. Field report. Refugees International (2015).

[51] USCIRF. Central African Republic. United States Commission on International Religious Freedom. Available at: http://www.uscirf.gov/sites/default/files/USCIRF_AR_2016_Tier1_2_CAR.pdf (2016).

[52] Hyndman RJ, Koehler AB (2006). Another look at measures of forecast accuracy. International Journal of Forecasting.

[53] Moore WH, Shellman SM (2006). Refugee or internally displaced person? To where should one flee?. Comparative Political Studies.

[54] UNHCR. Global report. United Nations High Commissioner for Refugees. Available at: http://www.unhcr.org/gr15/index.xml (2015).

