# Supplementary material for: A generalized simulation development approach for predicting refugee destinations
Source: Sci Rep. 2017 Oct 17;7:13377. doi: 10.1038/s41598-017-13828-9 (PMC5645318; doi:10.1038/s41598-017-13828-9)
Supplement: Supplementary file 1 — Supplementary Information [file 41598_2017_13828_MOESM1_ESM.pdf]

Supplementary Information for ”A generalized simulation development approach for predicting refugee destinations”

Diana Suleimenova[1], **David Bell**[1], **and Derek Groen**[1,2,*]

[1]Brunel University London, Computer Science, London, UB8 3PH, United Kingdom
[2]University College London, Centre for Computational Science, London, WC1H 0AJ, United Kingdom.
Correspondence and requests for materials should be addressed to D.G. (email: derek.groen@brunel.ac.uk)

ABSTRACT

This document serves as Supplementary Information for the paper ”A generalized simulation development approach for predicting refugee destinations”

Supplementary Note 1. Detailed background information on the three African conflicts

Here we present a generalized prediction approach for simulation refugee movements, and test the validity of our approach by comparing it with empirical data from three separate conflicts in Africa. These conflicts are the 2015 civil war in Burundi, the conflict in the Central African Republic between 2013 and 2016, and the Northern Mali conflict in 2012.

Burundi has a lengthy history of civil war resolved with the 2000 Arusha Peace and Reconciliation Agreement. The post-war position of the country supported and focused on power sharing constitution leading to peace and progress. Pierre Nkurunziza, who was the Chairman of the National Council for the Defence of Democracy-Forces for the Defence of Democracy (CNDD-FDD) party, was elected as the first president. Despite an increasing concern of corruption and autocratic governing, Nkurunziza continued for the second presidential term. The Arusha Agreement encloses a two term limit, however, in April 2015 the CNDD-FDD anew elected the first president for the third term[1,2]. This has triggered protests, coups and refugee crisis forcing people to flee to the neighbouring countries of Rwanda, Uganda, Tanzania and the Democratic Republic of Congo (DRC). Each neighbouring country has several registration locations and camps were refugees allocated[3]. Hence, we adopted the current situation of Burundi for the simulation starting from the 1st May 2015 until 31st May 2016. Burundi simulation scenario has 8 major conflict locations, starting in capital Bujumbura from the start of the simulation and including other province capitals, namely, Cibitoke, Bubanza, Kayanza and other cities.

In March 2013, the central government of the Central African Republic (CAR) were overthrown by the Seleka group, which represents mainly Muslim population located in the north of the country. However, not long after anti-Balaka, who are Christian militia groups took over the power from the Seleka group. This has enhanced the conflicts and violent attacks between communities of Muslim and Christians. Subsequently, other communities, such as agriculturalists associated with anti-Balaka and Muslim herders linked with ex-Seleka, increased clashes in CAR. With the escalation of fighting, the situation in the country became unstable and dangerous resulting in forced population displacement internally and to neighbouring countries, namely Chad, Cameroon, DRC and the Republic of Congo (RC). The situation of CAR adopted for the simulation analysis using time period from 1st December 2013 to 29th February 2016.

In Mali we focus on the Northern Mali Conflict that took place in 2012, when insurgent groups began a campaign to fight for independence of the Azawad region in Northern Mali. This situation is described in detail by Groen[4]. The Northern Mali conflict started on January 16th 2012, when Touareg rebels began conquering places in Northern Mali, and the simulations start on February 29th, the data that the first refugee registrations were done. This scenario has three major conflict locations, starting in Kidal from the beginning of the simulation, and adding Gao and Timbuktu on the 31st of March (day 31). The total number of refugees registered in these camps reaches a peak of $\sim$145,000 around day 170.

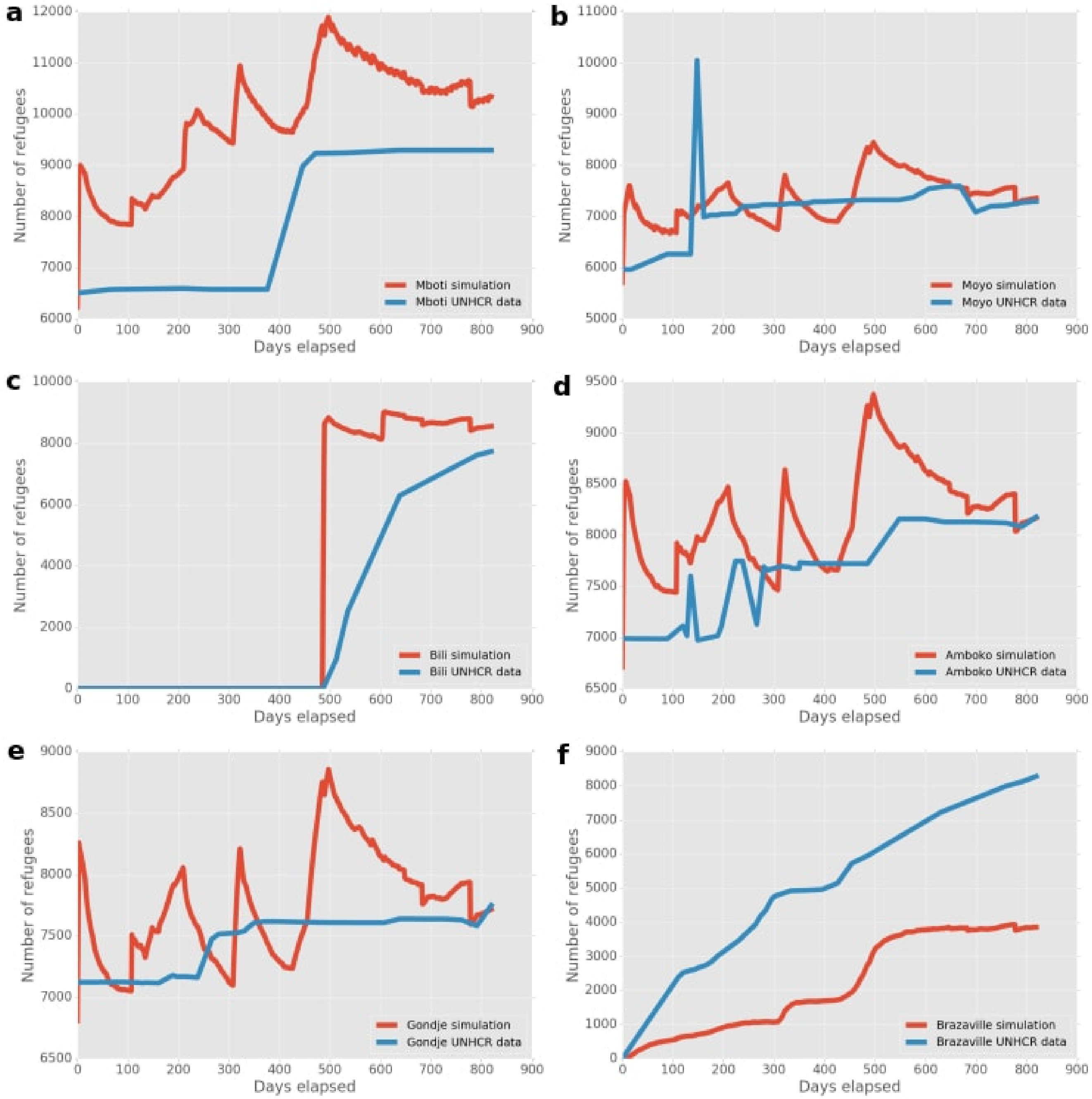

Figure 1. Relative fraction of refugees as predicted by our simulation and obtained from the UNHCR data for the CAR conflict. **(a-f)** Graphs are ordered by camp population size, with the most populous camp on the top to the smallest one on the bottom.

Supplementary Note 2. Source data pertaining to the three simulations

We derive our conflict events from the Armed Conflict Location and Event Data Project (acleddata.com), selecting each ”Battle” event from the database. Whenever such an event initially occurs, we mark the selected location as a conflict zone. The occurrence of subsequent events do not alter the status of the location, as locations remain a conflict zone for the duration of the simulation once they have been marked as such. A list of the initial conflict events can be found in Table 1 for Burundi, in Table 2 for CAR, and Table 3 for Mali.

Table 1. Burundi: Occurrence of conflicts

Date	Day in simulation	Conflict Location
1 May 2015	1	Bujumbura
10 July 2015	70	Kabarore
11 July 2015	71	Bukinanyana
15 July 2015	75	Cibitoke
26 October 2015	178	Mwaro
23 November 2015	206	Gisuru
8 December 2015	221	Burambi

Table 2. Central African Republic: Occurrence of conflicts

Date	Day in simulation	Conflict location
10 December 2012	*	Ndele
15 December 2012	*	Bamingui
28 December 2012	*	Bambari
18 January 2013	*	Obo
11 March 2013	*	Bangassou
24 March 2013	*	Bangui
17 April 2013	*	Mbres
3 May 2013	*	Bohong
17 May 2013	*	Bouca
7 September 2013	*	Bossangoa
14 September 2013	*	Bossembele
10 October 2013	*	Bogangolo
26 October 2013	*	Bouar
10 November 2013	*	Rafai
28 November 2013	*	Damara
6 December 2013	5	Bozoum
1 January 2014	31	Bimbo
28 January 2014	58	Boda
6 February 2014	67	Kaga-Bandoro
11 February 2014	72	Berberati
11 March 2014	100	Nola
8 April 2014	128	Dekoa
10 April 2014	130	Bria
14 April 2014	134	Grimari
26 April 2014	146	Paoua
23 May 2014	173	Carnot
30 July 2014	241	Batangafo
10 October 2014	313	Sibut

* Conflict zones that occurred before simulation period

Table 3. Mali: Occurrence of conflicts

Date	Day in simulation	Conflict Location
3 February 2012	*	Kidal and Timbuktu
29 February 2012	1	Menaka
2 March 2012	2	Tenenkou
13 March 2012	13	Dire
23 March 2012	23	Gao
30 March 2012	30	Bourem and Ansongo
10 August 2012	163	Bamako
1 September 2012	185	Douentza
28 November 2012	273	Lere

*Conflict zones that occurred before simulation period

In addition, a number of camps open after each conflict has commenced, as the UNHCR data indicate no influx of refugees there prior to a certain date. These include:

- Lusenda (Burundi) on July 30th, 2015.
- Nduta (Burundi) on August 10th, 2015.
- Bili (CAR) on April 1st, 2015.

Also, refugees were redirected to camps from a range of locations in the same country, according to a range of UNHCR reports. These locations include:

- Nduta, forwarding to Nyarugusu (Burundi, prior to August 10th, 2015).
- Kagunga, forwarding to Nyarugusu (Burundi).
- Gado-Badzere, forwarding to East (CAR).
- Lolo, forwarding to East (CAR).
- Mbile, forwarding to East (CAR).
- Timangolo, forwarding to East (CAR).
- Borgop, forwarding to Ademaoua (CAR).
- Ngam, forwarding to Ademaoua (CAR).
- Fassala, forwarding to Mbera (Mali).

We implemented the above locations as forced redirection points, which means that all refugees arriving in this location immediately continue their journey towards the destination. Most of these forwarding points reside within Cameroon in the CAR simulation. Here, refugees tend to be scattered across the region, and the refugee count is aggregated for the East and Ademaoua regions. To reflect this, we placed the smaller camps within the simulation graph, but forwarded any refugees arriving at these smaller camps directly to the main locations.

Lastly, our simulations were subject to a range of reported border closures. These include:

- CAR - DRC Congo (from the 5th of December 2013 until the 30th of June 2014[5].
- CAR - Chad (from the 12th of May 2014 onwards[6].
- Mali - Burkina Faso (until the 21st of March 2013[7]).
- Mali - Niger (until the 1st of April 2013[8]).

Supplementary Note 3. Comparison with non-rescaled simulations

Here we present a comparison of the averaged relative difference in our simulation, both with and without rescaling, to provide a clear view to the reader how the rescaling affects the sensitivity of the simulation to different circumstances and settings. More details on how we do the rescaling correction can be found in the Methods section of the main manuscript (choosing simulation parameters and assumptions).

We present the averaged relative difference between our simulations and the UNHCR data values, both for our default simulations and for simulations that do not have the refugee counts rescaled, in Fig. 2. For the simulations of Burundi and Mali, we see a clear reduction in the averaged relative difference throughout the simulation when we rescale the refugee counts, whereas the difference is negligible for the simulation of the Central African Republic. Because this rescaling can be done without modifying our underlying data constraints and assumptions of our approach, and a clear benefit is visible in two of the three simulations, we chose to present our results in the main paper with rescaling enabled.

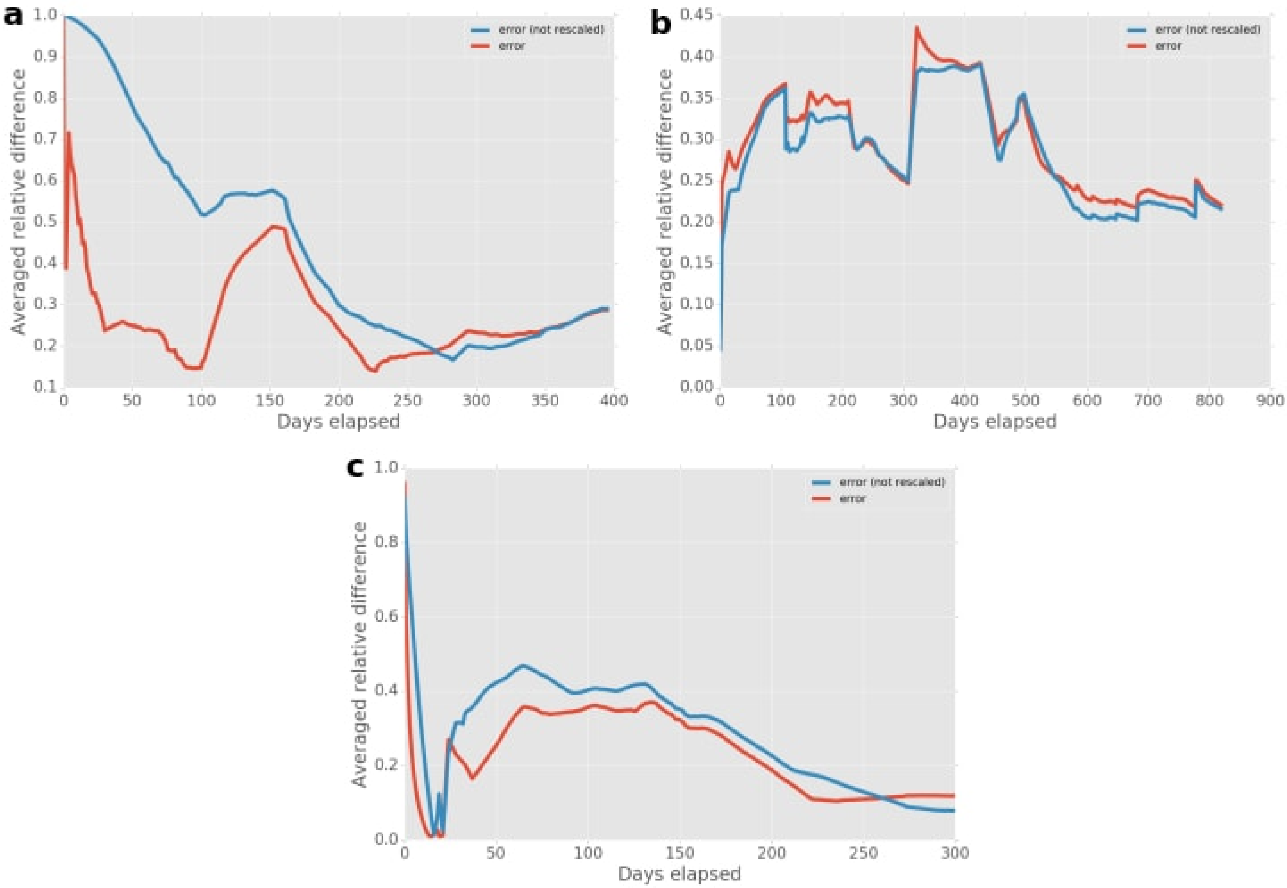

Figure 2. Overview of the averaged relative difference between simulation and data. The averaged relative difference across camps between simulation and data is given by the red line for rescaled simulations, and by the blue line for non-rescaled simulations. We provide these comparisons respectively for (a) the Burundi simulations, (b) the CAR simulations and (c) the Mali simulations.

Supplementary Note 4. Sensitivity testing

We performed a range of sensitivity tests, using simulations with rescaling enabled (as in the main paper), and with rescaling disabled. In total we have performed 435 simulations to investigate the sensitivity of various aspects in our approach, and to characterize uncertainty. This includes 300 simulations to test the variability between instances when using identical parameters (as our simulation has stochastic components), 24 simulations (8x3) to test the accuracy across different movement rate limits, 15 simulations to test the accuracy across different awareness ranges, 30 simulations to test the accuracy across different attractiveness values, and 66 simulations to test the accuracy across different move probability values.

Variability of runs using identical settings. Using awareness level 1 and a refugee movement speed of up to 200km/day, we ran 50 identical simulations of each situation to determine the variability in our error measure due to the randomized elements of our simulations. We present an overview of these variations in Tab. 4. Here we find that for rescaled simulations (the ones for which we present the results in the paper), the 95% confidence interval of the averaged relative difference is within 0.001 of the obtained averaged relative difference value.

Table 4. 95% confidence interval of the averaged relative difference across runs for the three conflict simulations, each of which have been run 50 times in total. The confidence interval provided only incorporates the uncertainty caused by the stochastic nature of our ABM algorithm, with all other parameters kept constant. We present runs for non-rescaled simulations (top three rows), as well as for different instances of the rescaled simulations that we present in the main paper (bottom rows).

**run name**	**average**	**confidence (min)**	**confidence (max)**	**confidence(max) - average**
Burundi (non-rescaled)	0.439610308771	0.439452241265	0.439768376276	0.000158067505508
CAR (non-rescaled)	0.282915499648	0.282597354563	0.283233644733	0.000318145085201
Mali (non-rescaled)	0.288257106577	0.287878843721	0.288635369433	0.000378262855805
Burundi (rescaled)	0.280668715754	0.279672757258	0.28166467425	0.00099595849637
CAR (rescaled)	0.295634817802	0.295317651166	0.295951984437	0.000317166635253
Mali (rescaled)	0.231155570689	0.230756281742	0.231554859635	0.000399288946948

Comparison of simulation accuracy across different movement rate limits for refugees. We are able to adjust the maximum movement speed of refugee agents within our model. Refugees will never move faster than this limit, but will move slower for example when agents stop at an intermediate (non conflict-zone) locations. We present the averaged relative difference of our simulation as a function of this parameter in Table 5. Increasing this limit to larger values reduces the error in non-rescaled simulations, because it tends to reduce the mismatch between total number of encamped refugees in the simulation, and in the UNHCR data set. Please note that we place refugees in conflict areas on the day that they are registered in camps in the UNHCR data, the departure dates of the refugees is not contained in the dataset.

However, when we look at rescaled simulations, we find that the averaged relative difference decreases only up to a point when we increase the movement rate limit. Beyond a limit of 200 km/day we do not observe any additional accuracy benefits. For the simulations in our main paper, we therefore use a maximum refugee movement speed of 200 km/day.

Table 5. Averaged relative difference for each simulation using different speed limits of refugees, with an awareness range of 1 link away. Averaged relative difference values, averaged over all three types of simulations, without rescaling can be found in column 2, and values with rescaling in column 3. The agent-based simulations presented in the main paper have a maximum speed of 200 km/day.

Maximum refugee speed	Averaged relative difference	
[km/day]	**Not rescaled**	**Rescaled**
25	0.448361226379	0.356560873468
50	0.394881861148	0.312680106688
100	0.353853333946	0.279609237892
150	0.34318164792	0.273509521162
200 (default)	0.336953617616	0.266171711355
250	0.333588139275	0.266402739391
500	0.32526508617	0.263586214986
1000	0.323284911458	0.261094328949

Comparison of simulation accuracy across different levels of agent awareness. Within our simulation model, we are able to adjust our algorithm to incorporate a more wide or narrow awareness level for the agents. We present the averaged relative

difference between our simulations and the UNHCR data as a function of different awareness levels in Table 6. Agents can be aware of the presence of paths only (”Unweighted”), of only the length of the path to the nearest settlement (”Path distance only”), the type of nearest settlement (”1 link away”), the type of settlements adjacent to neighbouring settlements (”2 links away”), and the type of settlements neighbouring those neighbours of neighbours (”3 links away”). The agent-based simulations presented in the main paper have an awareness range which stretches up to locations 1 link away from their current position, and use rescaling.

Table 6. Averaged relative difference for each simulation using different levels of agent awareness range. Averaged relative difference values, averaged over all three types of simulations, without rescaling can be found in column 2, and values with rescaling in column 3.

	Averaged relative difference	
Awareness range	**Not rescaled**	**Rescaled**
Unweighted	0.356858849696	0.341601692112
Path distance only	0.340677121531	0.266356996267
1 link away	0.336953617616	0.266171711355
2 links away	0.325889236988	0.267601141905
3 links away	0.32397048081	0.26248797001

Comparison of simulation accuracy across different attractiveness values for camps and conflict zones. Within our simulation model, we are able to adjust our algorithm to increase or decrease the attractiveness value for camp locations, as well as for conflict zones. We present the averaged relative difference between our simulations and the UNHCR data as a function of the different camp attractiveness values in Table 7, and as a function of the different conflict attractiveness values in Table 8. For the rescaled simulations, we find that the averaged relative difference varies by less than 0.008 throughout the range of tested parameter values. As a result, the quality of our validation is relatively insensitive to the choice of these two parameters.

Table 7. Averaged relative difference for each simulation using different weight multipliers for camps in agent destination selection (with a value of 2.0 making camps twice as likely to be chosen as destination compared to other locations). Values without rescaling can be found in column 2, and values with rescaling in column 3.

	Averaged relative difference	
Camp weight multiplier	**Not rescaled**	**Rescaled**
1.5	0.338927391256	0.265446181807
1.75	0.337844532212	0.265843514126
2 (default)	0.336573946993	0.26880034312
2.25	0.336573297016	0.270188679407
2.5	0.336645040133	0.272965319355

Table 8. Averaged relative difference for each simulation using different weight multipliers for conflict zones in agent destination selection (with a value of 0.25 making conflict zones four times less likely to be chosen as destination compared to other locations). Values without rescaling can be found in column 2, and values with rescaling in column 3.

	Averaged relative difference	
Conflict zone weight multiplier	**Not rescaled**	**Rescaled**
0.15	0.343139359501	0.276699204298
0.2	0.340114654169	0.272082272463
0.25 (default)	0.337231546257	0.26999847858
0.3	0.334752333474	0.265096045458
0.35	0.33401435684	0.26418701977

Comparison of simulation accuracy across different move probabilities for agents. Within our simulation model, we are able to adjust our algorithm to increase or decrease the probability that agents move from their current location to a different one on a given day. This probability is set according to the type of location where an agent resides, which is either a conflict zone, camp, or other (default) location. We present the averaged relative difference between our simulations and the UNHCR data as a function of the different conflict zone move probabilities in Table 9, and as a function of the different camp move probabilities

in Table 10. For the rescaled simulations, we find that the averaged relative difference varies by less than 0.002 throughout the range of tested parameter values for conflict move probabilities and that the quality of our validation is relatively insensitive to the choice of these two parameters. However, our simulation results are somewhat sensitive to camp move probabilities, where a move probability of 0.05 (i.e., a refugee agent will remain in a camp for 20 days on average, rather than 1000 days) will lead to an increase in error of 0.03. Likewise, reducing this parameter to 0.00001 (i.e., a refugee agent will remain in a camp for 100000 days on average) will lead to an error increase of approximately 0.01.

Table 9. Averaged relative difference for each simulation using different move probabilities for agents which reside in conflict zones (default=1.0). Values without rescaling can be found in column 2, and values with rescaling in column 3.

	Averaged relative difference	
Run type	**Not rescaled**	**Rescaled**
1.0 (default)	0.336989892104	0.269482844178
0.95	0.337022779555	0.269168207032
0.9	0.336684880025	0.269283340368
0.85	0.336209055377	0.268405759495
0.8	0.337617947407	0.270178424251

Table 10. Averaged relative difference for each simulation using different move probabilities for agents which reside in camps (default=0.001). Values without rescaling can be found in column 2, and values with rescaling in column 3.

	Averaged relative difference	
Run type	**Not rescaled**	**Rescaled**
1e-05	0.339436280234	0.278773614336
0.0001	0.339043699152	0.278447962132
0.001 (default)	0.337477094236	0.269408281682
0.01	0.349965762497	0.266830566912
0.05	0.40924478076	0.298998969716

We present the averaged relative difference between our simulations and the UNHCR data as a function of the different default move probabilities in Table 11. The default move chance is the main parameter that determines how frequently refugee agents move away from their current location towards an adjacent one. For the rescaled simulations, we find that our simulation results are sensitive to the value of this parameter, with lower values leading to a higher error (up to 0.026 higher with move probability 0.2), and higher values leading to a smaller error (up to 0.029 lower with move probability 1.0). The errors for the non-rescaled simulations decrease more drastically with this move probability, with an error of 0.378 for probability value 0.2 and 0.253 for probability value 1.0.

Table 11. Averaged relative difference for each simulation using different default move probabilities for agents which reside in locations other than camps and conflict zones (default=0.3). Values without rescaling can be found in column 2, and values with rescaling in column 3.

	Averaged relative difference	
Run type	**Not rescaled**	**Rescaled**
0.2	0.377890468924	0.293764070066
0.25	0.355661461333	0.279774321428
0.3 (default)	0.336759969188	0.267795549495
0.35	0.32370928651	0.263387529768
0.4	0.311404558564	0.257848585117
0.45	0.301036733529	0.253167138021
0.5	0.292766862664	0.250971365696
0.6	0.278225342605	0.24666371555
0.7	0.268040877633	0.242262185396
0.8	0.260984597208	0.240733898578
0.9	0.255783141742	0.239805668192
1.0	0.252630160883	0.238616738835

There are multiple possible explanations for these results. For example, it is possible that our initial assumption (refugees have a chance of 0.3 on any given day to depart a non-conflict location in the country of conflict, and therefore stay in such a place for an average duration of 3.33 days) was an underestimation, and that refugees tend to move from place to place much more frequently. However, it is also possible that a higher value for this parameter is unrelated to the accuracy of our associated assumption, and that the error becomes lower simply because the mismatch in refugee arrivals between simulation and data is artificially reduced (see Methods section, subtopic "Choosing simulation parameters and assumptions"). The observed narrowing of the gap between the non-rescaled and rescaled errors in the results (from 0.084 for probability 0.2 to 0.014 for probability 1.0) could be explained by this latter theory.

Supplementary Note 5. Summary of differences between the Mali simulation in this paper and that of the 2015 ICCS publication

We previously used a prototype version of FLEE to perform a proof-of-concept simulation of refugee movements in Mali[4]. Here we provide a summary of the main differences between the approach we have adopted here, and the simulation we performed in this earlier work. We provide a listing of the key differences in Table 12. Because we did not yet incorporate mismatches between level 1 and level 2 registrations in the data, we compared our simulation results against uncorrected UNHCR data in the ICCS paper. This can be clearly observed in Figure 2j of that paper, were the number of refugee agents appears to decrease steeply in the latter half of the simulation. In reality, this perceived drop is due to mismatches between level 1 and level 2 registration counts (as mentioned in the main paper). Due to the presence of these mismatches, the reported averaged relative difference in the ICCS paper is not directly comparable to the reported averaged relative differences in this paper.

Table 12. Key differences in the Mali simulation as constructed and performed here, and the one performed in the 2016 ICCS paper[4].

Aspect of simulation construction	Now	2016 ICCS paper
Number of conflict locations in simulation	11	3
Source of conflict information	ACLED	Wikipedia
Number of locations in Mali	16	9
Number of locations (total)	24	17
Correct data for mismatch between level 1 and level 2 registrations	Yes	No
Awareness range	1 link away	path distance only
FLEE version	1.0	0.1b (non-public beta)

Supplementary Note 6. A description of the basic assumptions of the algorithm used by FLEE

We present a flowchart description of the algorithm assumptions used by FLEE code in the Figure 3 and 4 discussed in detail in Methods section of the main paper.

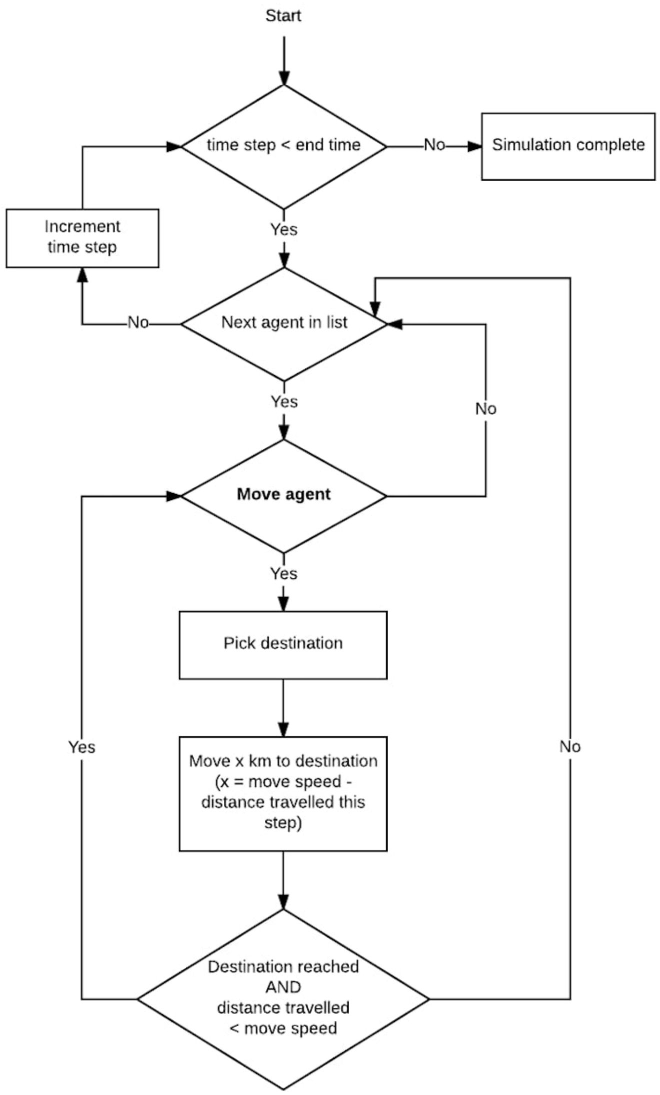

Figure 3. A basic flowchart of algorithm assumptions used by FLEE code to predict refugee destinations. Move agent factor has a detailed sub-path demonstrated in Figure 4.

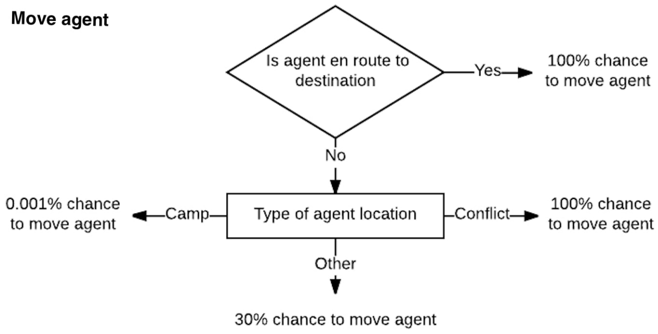

Figure 4. A detailed description of move agent where agents (refugees) have three variations of location determined by the move chances.

References

1. Boyce, M. & Vigaud-Walsh, F. 'You are either with us or against us': Persecution and displacement in Burundi. Refugees International (2015).
2. IRRI. Burundi: A country on the edge. International Refugee Rights Initiative. Available at: http://www.refworld.org/docid/57b6f9364.html (2016).
3. Raleigh, C., Kishi, R., McKnight, J. & Moody, J. Country report: Burundi crisis year one. Country report. Armed Conflict Location and Event Dataset (2016).
4. Groen, D. Simulating refugee movements: Where would you go? *Procedia Comput. Sci.* **80**, 2251–2255 (2016).
5. UNHCR. UNHCR regional update no. 1 CAR situation. United Nations High Commissioner for Refugees. Available at: http://data.unhcr.org/car/download.php?id=21.
6. Reuters. Chad closes border with Central African Republic. Reuters. Available at: http://www.reuters.com/article/us-centralafrica-chad-idUSBREA4B0M320140512.
7. UNHCR. UNHCR Mali report no. 3. United Nations High Commissioner for Refugees. Available at: http://www.unhcr.org/uk/publications/operations/4f7c49999/mali-situation-update-3-30-march-2012.html.
8. UNHCR. UNHCR Mali report no. 4. United Nations High Commissioner for Refugees. Available at: http://www.unhcr.org/uk/publications/operations/4f9952ce9/mali-situation-update-4-24-april-2012.html.
